# Laparoscopic Ureteral Substitution Using the Appendix for Complete Stenosis of the Middle Ureter due to Ureteral Stone in an Elderly Patient: A Case Report

**DOI:** 10.1002/iju5.70112

**Published:** 2025-11-09

**Authors:** Ryutaro Fukagai, Shuichi Shimabukuro, Masafumi Ie, Minoru Nakazono, Masashi Teramoto, Atsuhiko Ochi, Koichiro Suzuki, Akira Komiya, Hirokazu Abe

**Affiliations:** ^1^ Department of Urology Kameda Medical Center Chiba Japan; ^2^ Department of Urology Okinawa Prefectural Chubu Hospital Okinawa Japan; ^3^ Department of General Surgery Okinawa Prefectural Chubu Hospital Okinawa Japan

**Keywords:** appendix, elderly, laparoscopic surgery, ureteral stenosis, ureteral substitution

## Abstract

**Introduction:**

While established in pediatric urology, the laparoscopic use of appendiceal substitution of the ureter in adults is rare.

**Case Presentation:**

An 83‐year‐old man developed a 2.5‐cm right mid‐ureteral stricture as a complication after endoscopic treatment for a ureteral stone. Preoperative imaging confirmed an adequate appendix length. A laparoscopic approach was selected due to its minimal invasiveness, which was considered particularly appropriate for an elderly patient. After isolation with a linear cutting stapler, both ends of the appendix were resected, and a guidewire was passed through. To prevent mesoappendix torsion, the cecal side was oriented cranially before ureteral anastomosis. The operative time was 336 min, with 10 mL blood loss. Postoperative ureteral stone formation resolved spontaneously, and renal function was preserved.

**Conclusion:**

Laparoscopic appendiceal interposition is a viable and minimally invasive option for right mid‐ureteral reconstruction in selected elderly patients.

**Abbreviations**
CT: computed tomography
MRI: magnetic resonance imaging
TUL: transurethral ureterolithotripsy

**Summary**

- Laparoscopic appendiceal interposition is rare but effective for reconstructing mid‐ureteral defects.
- In our case, an elderly patient was subjected to minimally invasive surgery using the appendix to bridge a ureteral stricture, avoiding more invasive procedures and reducing surgical stress.
- The case highlights the clinical feasibility of using the appendix as a substitute ureter, even in older adults.

## 1 Introduction

Appendiceal substitution of the ureter is well‐described in pediatric urology but rarely performed in adults, particularly via laparoscopy. We present a case of laparoscopic appendiceal ureteral substitution in an elderly patient with complete right mid‐ureteral stenosis due to a ureteral stone.

## 2 Case Presentation

**FIGURE 1:**
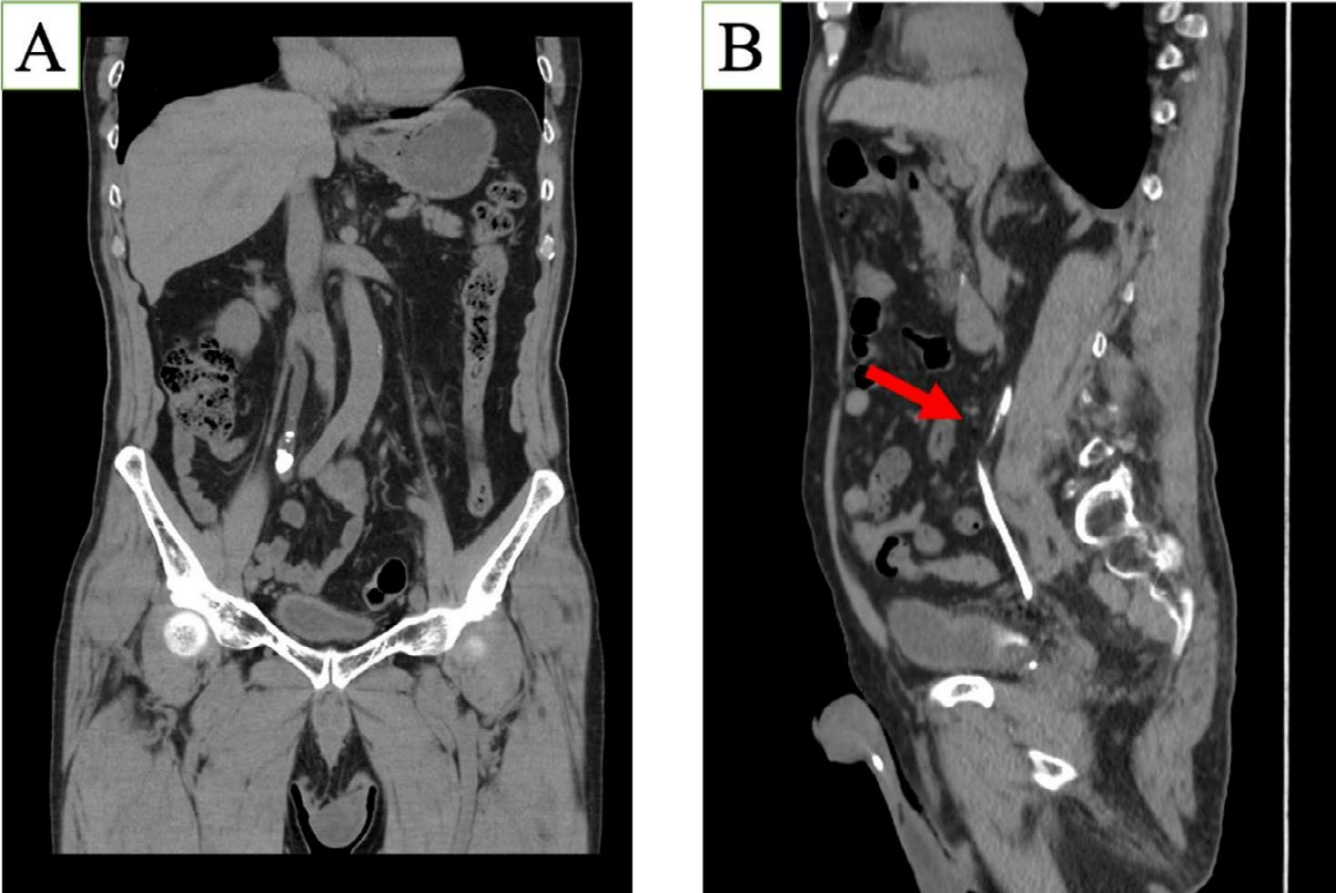
(A) Ureteral stone in the ureter. (B) Location of stenosis.

An 83‐year‐old man was referred to our institution for surgical management of a 2.5‐cm right mid‐ureteral stricture. He had undergone transurethral ureterolithotripsy (TUL) for a 13‐mm right ureteral stone at the same site 8 months prior (Figure 1A), but complete fragmentation was difficult, and a stent was placed. Two months later, TUL was attempted; however, due to severe stricture, the procedure was not feasible. A nephrostomy was subsequently placed, and antegrade ureteroscopy was attempted. During this procedure, a flexible ureteroscope was inserted from the nephrostomy route, and the stone was successfully fragmented and retrieved. Nevertheless, the guidewire could not be passed due to persistent stenosis. Contrast‐enhanced investigation revealed a 2.5‐cm segmental narrowing located at the level of the fifth lumbar vertebra (L5) (Figure 1B). He was managed with a nephrostomy and monthly catheter changes, but expressed a strong desire for nephrostomy removal. At the time of presentation to our hospital, his serum creatinine was 1.48 mg/dL. A laparoscopic approach was selected due to sufficient appendix length and a favorable preoperative evaluation.

## 3 Surgical Methods and Procedures

The patient was placed in the lithotomy position with a slight leftward head‐down tilt. The surgeon stood on the left side, while the assistant was positioned cephalad on the same side.

**FIGURE 2:**
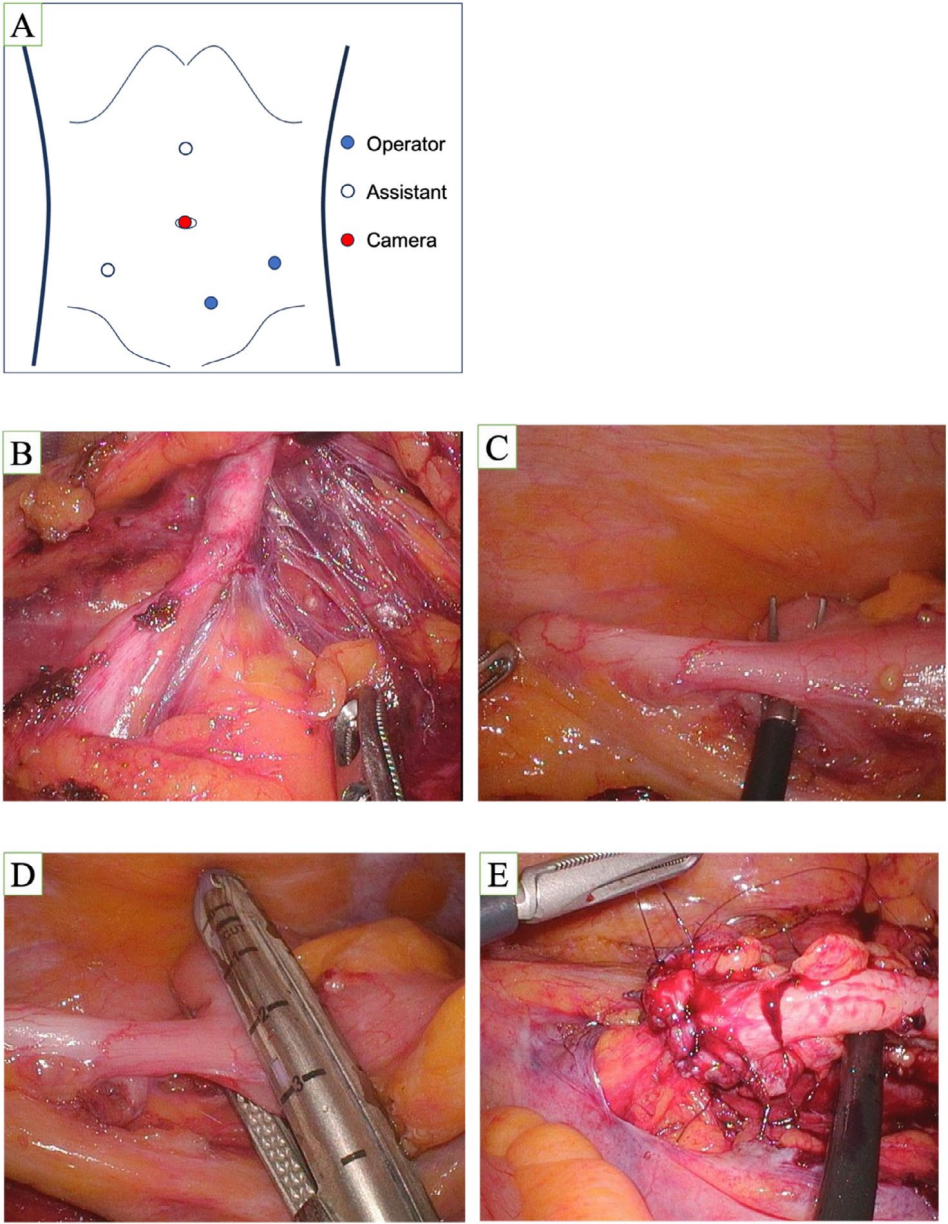
(A) Placement of the camera port and assistant port in the operation. (B) Dissection of the ureter. (C) Exposed appendix. (D) Separation from the mesentery with a GIA stapler. (E) Anastomosis of the caudal side with 4‐0 absorbable monofilament sutures.

A ureteral catheter was inserted via cystoscopy and fixed to a bladder catheter. Ports were placed: a 12‐mm camera port at the umbilicus, two 5‐mm ports 8 cm cephalad and caudad, and two 5‐mm ports medial to the anterior superior iliac spines. The caudal port was slightly left of midline (Figure 2A). The peritoneum was incised from the right common iliac artery to the ileocecal region to access the retroperitoneum. The ureter was identified medial to the gonadal vein and dissected 2.5 cm above and below the stricture.

The stenotic segment was located by direct visualization (Figure 2B) and confirmed based on the limited mobility during catheter manipulation and tactile stiffness when grasped with forceps. The appendix was mobilized and transected using a linear cutting stapler (Figure 2C,D). Both ends were trimmed, and a guidewire was passed through. The cecal side was positioned cranially to avoid torsion. A 5‐mm incision was made in the healthy ureter, and end‐to‐end anastomosis was performed using 4‐0 absorbable monofilament sutures (Figure 2E). A 6Fr ureteral stent and a drain were placed. The operative time was 336 min, and estimated blood loss was 10 mL.

## 4 Postoperative Clinical Course

**FIGURE 3:**
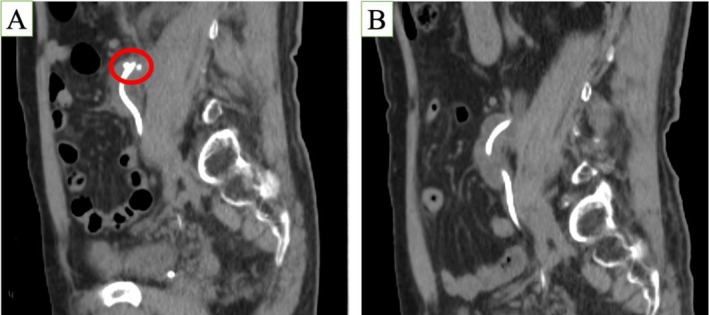
(A) A ureteral stone was found in the upper side of the anastomosis. (B) Computed tomography on postoperative Day 181 showed no stones.

**FIGURE 4:**
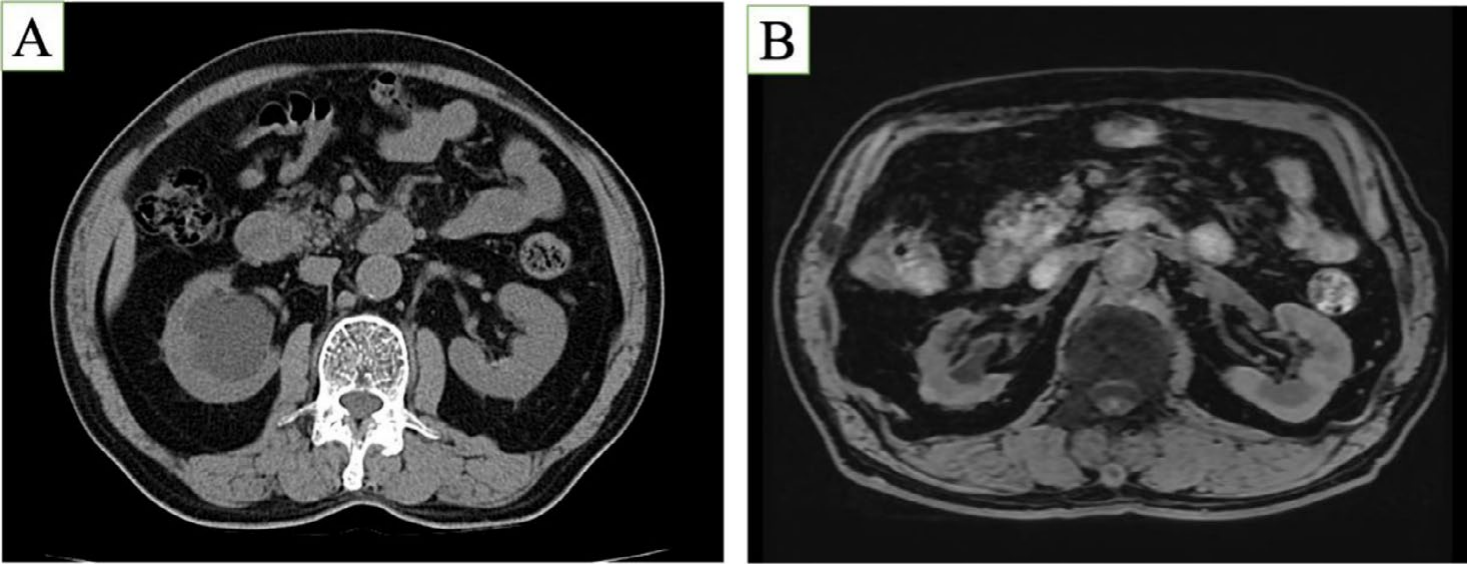
(A) Hydronephrosis before surgery. (B) On Day 226, magnetic resonance imaging indicated improved hydronephrosis.

The drain was removed on postoperative Day 2, and the nephrostomy on Day 25. Computed tomography (CT) on Day 33 revealed a stone above the anastomosis (Figure 3A) and hydronephrosis. TUL was attempted on Day 85, but a 10 Fr dilator could not pass; only a stent exchange was performed. CT on Day 181 showed spontaneous stone passage (Figure 3B), and the stent was removed. Magnetic resonance imaging (MRI) on Day 226 confirmed improvement in hydronephrosis and preserved renal function (Figure 4A,B).

## 5 Discussion

Several options for the reconstruction of mid‐ureteral defects are available, including ureteroureterostomy, renal auto transplantation, and the use of bowel segments, such as ileal ureter [1]. In this case, we selected appendiceal interposition. Preoperative contrast‐enhanced CT confirmed sufficient appendix length spanning the 2.5‐cm defect. The absence of prior abdominal surgery supported a laparoscopic approach using the appendix due to expected ease of mobilization and low adhesion risk. Although this technique involves bowel tissue, it does not require intestinal‐to‐intestinal anastomosis, which is necessary in ileal ureter substitution, thereby reducing the overall surgical invasiveness.

Other options, such as buccal mucosa grafting or ileal ureter substitution, were considered, but the appendix offered key advantages. Compared to buccal mucosa, which requires technically demanding laparoscopic tubularization and finer suturing, the appendix is inherently tubular and easier to handle laparoscopically. Additionally, unlike bowel segments, the appendix does not reabsorb urine, reducing the risk of electrolyte imbalance, which is an important consideration in elderly patients. If the appendix was short or unusable due to inflammation or adhesions, ureteroureterostomy after renal descent or Yang‐Monti ileal ureter could be considered.

Appendiceal use in urinary tract reconstruction was first described by Mitrofanoff in 1912 as a catheterizable channel in children [2]. Its use as a ureteral substitute has since been reported in pediatric patients with favorable long‐term outcomes [3]. Cases in adults, though rare, have been described [4], including successful laparoscopic applications [5], but are extremely limited in Japan due to technical complexity. Only a few cases of laparoscopic appendiceal ureteral substitution in adults have been reported to date, including four described by Komyakov et al. [5]. No such case has been reported in Japan.

To preserve blood flow and avoid mesoappendix torsion, the cecal side of the appendix was anastomosed to the proximal ureter, as described previously [6]. One report described an anastomosis in which the appendiceal tip was connected to the proximal ureter, based on the authors' assumption that the appendix exhibits inherent peristaltic movement from the tip toward the base [4]; however, there is no clear consensus on this function. If such peristalsis exists, anastomosis may oppose urinary flow and potentially impair drainage or contribute to stone formation. However, favorable outcomes have been reported using the same orientation. Considering the risk of perfusion compromise from twisting the mesoappendix, we believe orientation should be determined on a case‐by‐case basis, considering anatomy, blood supply, and spatial relationship.

In this case, the procedure was performed laparoscopically, but if performed robotically, the anastomosis would likely be easier, and the surgical burden on the patient would be reduced. For these reasons, this method should be considered a viable option when appropriately indicated.

## 6 Conclusion

We successfully performed laparoscopic appendiceal interposition for a 2.5‐cm mid‐ureteral stricture. This technique is a viable and minimally invasive option for ureteral reconstruction, particularly in carefully selected elderly patients.

## Ethics Statement

The authors have nothing to report.

## Consent

Informed consent was obtained from the patient for the publication of this article and the accompanying images.

## Conflicts of Interest

The authors declare no conflicts of interest.

## Data Availability

The data that support the findings of this study are available on request from the corresponding author. The data are not publicly available due to privacy or ethical restrictions.
